# The Added Value of Controlling Nutritional Status (Conut) Score for Preoperative Counselling on Significant Early Loss of Renal Function After Radical Nephrectomy for Renal Cell Carcinoma

**DOI:** 10.3390/cancers16203519

**Published:** 2024-10-17

**Authors:** Matteo Boltri, Fabio Traunero, Luca Ongaro, Francesca Migliozzi, Fabio Vianello, Oliviero Lenardon, Francesco Visalli, Lorenzo Buttazzi, Daniele Maruzzi, Carlo Trombetta, Alchiede Simonato, Nicola Pavan, Francesco Claps

**Affiliations:** 1 Urological Clinic, Department of Medical, Surgical and Health Sciences, University of Trieste, 34127 Trieste, Italy; matteo.boltri@gmail.com (M.B.); fabio.tra92@gmail.com (F.T.); francesca.migliozzi@asugi.sanita.fvg.it (F.M.); trombcar@units.it (C.T.); nicpavan@gmail.com (N.P.); 2 Urology Unit, “San Giovanni di Dio” Hospital, 34170 Gorizia, Italy; fabio.vianello@asugi.sanita.fvg.it; 3 Department of Urology, Royal Free London NHS Foundation Trust, London NW3 2QG, UK; 4 Urology Department, “Santa Maria degli Angeli” Hospital, 33170 Pordenone, Italy; oliviero.lenardon@asfo.sanita.fvg.it (O.L.); francesco.visalli@aulss2.veneto.it (F.V.); buttazzilorenzo@hotmail.com (L.B.); daniele.maruzzi@gmail.com (D.M.); 5 Department of Surgical, Oncological and Stomatological Sciences, University of Palermo, 90133 Palermo, Italy; alchiede@gmail.com

**Keywords:** biomarkers, CONUT, morbidity, nutritional status assessment, chronic kidney disease, radical nephrectomy, Renal Cell Carcinoma

## Abstract

**Simple Summary:**

Renal Cell Carcinoma is a common cancer in Western countries. To date, the gold standard treatment for localized non-metastatic disease is partial or radical nephrectomy. When surgical treatment is feasible, the prediction of postoperative renal function might influence the treatment algorithm. On the other hand, there is growing evidence indicating that immune response and nutritional status are crucial factors in human cancer development and progression. In this scenario, the Controlling Nutritional Status (CONUT) score was initially developed as a malnutrition screening tool. Its formula includes serum albumin levels, total lymphocyte count, and total serum cholesterol. Serum albumin is used as an indicator of protein reserves. Cholesterol is used as a caloric depletion parameter. Total lymphocyte count represents an indicator of immune defense impairment due to malnutrition. In this study, we evaluated the impact of the CONUT score on clinically significant decline in estimated glomerular filtration rate in patients with non-metastatic Clear Cell Renal Cell Carcinoma undergoing radical nephrectomy. Our findings confirmed that a preoperative high CONUT score is an independent predictor of a significant renal function decline after surgery. Being easy to use, cost effective, and ideally a completely automated tool, its preoperative assessment could be part of a personalized risk-stratification tailored to the clinical conditions and comorbidities of each patient.

**Abstract:**

Background and Objectives: We aimed at evaluating the impact of Controlling Nutritional Status (CONUT) score on clinically significant decline in estimated glomerular filtration rate (eGFR) in patients with non-metastatic Clear Cell Renal Cell Carcinoma (ccRCC) undergoing radical nephrectomy (RN). Materials and methods: We retrospectively analyzed a multi-institutional cohort of 140 patients with ccRCC who underwent RN between 2016 and 2018 at three Urological Centers. The CONUT score was calculated with an algorithm including serum albumin, total lymphocyte count, and cholesterol. Clinical and pathologic features were analyzed using Fisher’s exact test for categorical variables and a Mann–Whitney U test for continuous variables. To define the independent predictors of clinically significant eGFR decline, univariable (UVA) and multivariable (MVA) binomial logistic regression analyses were performed in order to assess the Odds Ratio (OR) with 95% Confidence Intervals (CIs). Results: The optimal cut-off value to discriminate between a low and high CONUT score was assessed by calculating the ROC curve. The area under the curve (AUC) was 0.67 (95%CI 0.59–0.78) with the most appropriate cut-off value at 2 points. Overall, 46 patients (32.9%) had a high CONUT score (>2). Statistically significant variables associated with eGFR decline at 24 months were age ≥ 70 (OR 2.01; 95%CI 1.17–3.09; *p* = 0.05), stage II–III chronic kidney disease (CKD) (OR 6.05; 95%CI 1.79–28.3; *p* = 0.001), and a high CONUT score (OR 3.98; 95%CI 1.58–10.4; *p* = 0.004). Conclusions: The CONUT score is a low-time-consuming, cost-effective, and promising tool able to preoperatively screen patients at risk of developing CKD after a RN.

## 1. Introduction

Renal Cell Carcinoma (RCC) is a common disease, particularly among elderly and comorbid patients in Western countries, accounting for more than 431,000 cases and 179,368 deaths worldwide in 2020 [1]. It is frequently diagnosed as an incidental finding during abdominal ultrasound and cross-sectional imaging performed for other purposes [2]. To date, the gold standard treatment for localized non-metastatic RCC is partial or radical nephrectomy (PN-RN) performed with either a minimally invasive or open approach [3].

Surgical treatment may affect cancer-specific (CSS) and overall survival (OS) as much as the disease itself [4]. When surgical treatment is feasible, the prediction of postoperative renal function might influence the treatment algorithm [5]. Hence, a reliable and standardized preoperative counseling of mid- to long-term renal function impairment after surgery with curative intent is still an unmet clinical need.

There is growing evidence indicating that immune response and nutritional status are crucial factors in human cancer development and progression [6]. Therefore, the existing link between cancer and immuno-nutritional status has been a matter of study in recent decades [7]. In cancer patients, the risk for malnutrition is particularly high [7]. Accordingly, 10–20% of deaths in cancer patients can be attributed to malnutrition rather than to their primary malignancy [8].

Being a potentially modifiable condition, immune-nutritional status has gained growing interest in urological cancer patients [9,10,11]. In this scenario, the Controlling Nutritional Status (CONUT) was first developed in 2005 by de Ulíbarri et al. as a screening tool for malnutrition in inpatients’ setting [12]. The variables included in the CONUT formula were serum albumin levels (g/dL), total lymphocyte count (/mL), and total serum cholesterol (mg/dL). Serum albumin is used as an indicator of protein reserves. Cholesterol is used as a caloric depletion parameter. Total lymphocyte count represents an indicator of immune defense impairment due to malnutrition. Several retrospective studies have evaluated the prognostic significance of nutritional status in RCC patients undergoing RN or PN, considering recurrence-free survival (RFS), CSS, and OS [13,14,15,16]. Moreover, a significant prognostic impact of this index was further demonstrated in patients undergoing surgery for gastrointestinal (GI) malignancies [17,18].

To date, scarce evidence exists about the role of the CONUT score as a predictor of clinically significant decline in estimated glomerular filtration rate (eGFR) after RN for non-metastatic clear cell RCC (ccRCC). Hence, the objective of our study was to comprehensively evaluate the impact of preoperative nutritional status assessment provided by the CONUT score on the eGFR decline in RN candidates using individual patients’ data (IPD) of a multi-institutional collaboration. We hypothesized that impaired nutritional status as described by the CONUT score might be associated with a clinically significant eGFR decline after RN, potentially influencing survival outcomes.

## 2. Materials and Methods

### 2.1. Patients Characteristics

We retrospectively evaluated prospectively maintained data collected from 140 non-consecutive patients with computed tomography (CT)-diagnosed solid renal masses, suspicious for ccRCC, who underwent RN between 2016 and 2018 at three Italian Urological Centers. This study was conducted following the principles outlined in the Declaration of Helsinki. As per its retrospective observational non-interventional nature, this analysis was conducted on patients treated in accordance with the law and the national and European ethical guidelines. All of the authors ensured that their institutions and their clinical behavior are compliant with the specific requirements of the country. Informed consent for the use of personal data was regularly collected from all the subjects involved in this study. Signed informed consent forms are stored in an appropriate repository. Clinical Tumor Nodal Metastasis (cTNM) staging was preoperatively assessed with a total-body CT scan. Only cN0M0 patients were included. Variables collected included age, gender, Charlson Comorbidity Index (CCI), Body Mass Index (BMI), American Society of Anesthesiologists Classification (ASA) score, preoperative anemia, preoperative hypertension (HTN), Diabetes Mellitus (DM), eGFR calculated according to the Chronic Kidney Disease Epidemiology Collaboration (CKD-EPI) formula, pathological tumor (pT) and nodal (pN) stage, and Fuhrman grade. Surgical variables included approach and technique, intraoperative blood loss, intraoperative complication rate, and whether regional lymph node dissection (LND) was performed or not. Perioperative complications were reported according to the Clavien–Dindo classification, and further divided into minor (grade 1–2) and major complications (grade 3–5).

Preoperative blood samples were obtained within 30 days before surgery. The CONUT score was calculated using serum albumin concentration, peripheral lymphocyte count, and total cholesterol concentration [12] (Table 1). The preservation and handling of samples were conducted according to the national law and current technical regulations. RNs were performed by senior urological surgeons with either an open or laparoscopic/robotic approach at each center. The choice of surgical technique was made according to the surgeon’s preference. All RN specimens were locally reviewed by a dedicated uro-pathologist according to the standard guidelines [3]. Tumor grading was assessed according to the Fuhrman nuclear grading system. Patients were followed up according to European Association of Urology (EAU) RCC Guidelines at each participating center [3]. Follow-up consisted of medical history assessment, physical examination, serum chemistry, and radiological imaging as stated by EAU Guidelines. The postoperative eGFR assessment was performed on a regular basis. However, eGFR at 24 months was considered the reference value to define renal function loss after surgery, as it was available for all the patients and not influenced by peri-operative confounding factors such as the lack of an immediate compensation by the contralateral kidney [19]. Patients with incomplete follow-up or preoperative laboratory work-up were excluded.

### 2.2. Endpoints

Primary endpoint of the current analysis was a clinically significant eGFR decline defined as the development of a stage ≥ IIIb CKD (eGFR < 45 mL/min) at 24 months after surgery.

### 2.3. Statistical Analysis

Continuous variables were reported as medians with interquartile ranges (IQRs), while categorical variables were expressed as frequencies and proportions. The receiver operating characteristic (ROC) curve was obtained to determine the CONUT score optimal cut-off for the population, considering significant eGFR decline at 24 months as the endpoint of interest to yield the highest Youden Index. Patients were divided in two groups, namely a high (>2) and low (≤2) CONUT score. Clinical and pathological features were analyzed using Fisher’s exact test for categorical variables and a Mann–Whitney U-test for continuous variables. To define the independent predictors of clinically significant eGFR decline, univariable (UVA) and multivariable (MVA) binomial logistic regression analyses were performed to assess the Odds Ratio (OR) with 95% Confidence Intervals (CIs). Factors significantly influencing the eGFR decline development at UVA were further considered into the MVA model. Differences between the two groups were considered significant with a *p* < 0.05. An analysis was performed using R software version 4.0.5 (The R Foundation).

## 3. Results

### 3.1. Determination of CONUT Score and Cut-Off Value

**Figure 1:**
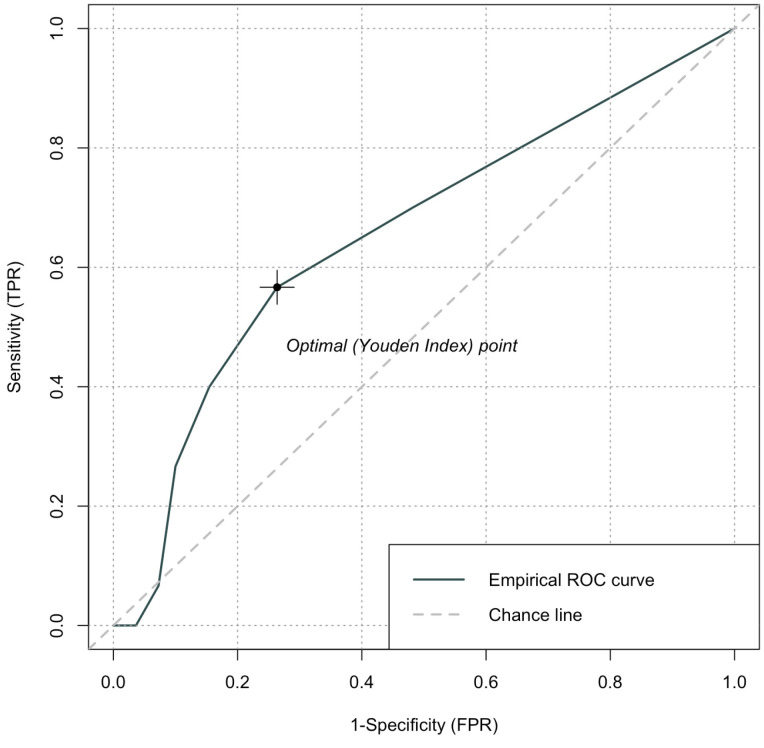
Receiver operating characteristic (ROC) curve for preoperative prediction of clinically significant eGFR decline defined as the development of a stage ≥ IIIb CKD (eGFR < 45 mL/min) at 24 months after RN. Abbreviations are as follows: eGFR: estimated glomerular filtration rate; CKD: chronic kidney disease; RN: radical nephrectomy.

The cut-off value to discriminate between a low and high CONUT score was defined by calculating the ROC curve. The area under the curve (AUC) was 0.67 (95%CI; 0.59–0.78) with the most appropriate cut-off value at 2 points (Figure 1). Patients were divided into low- (≤2) and high-CONUT-score groups (>2). A total of 94 patients were included in the former group and 46 patients in the latter, respectively.

### 3.2. Clinico-Pathological and Surgical Features of the Study Cohort

Table 2 summarizes the demographical and clinicopathological features of the two groups. Patients with a high CONUT score were significantly younger compared to their counterpart. Raised serum inflammatory markers (PCR and Fibrinogen) were prevalent in the high-CONUT-score group. Study populations were balanced in regard to cT stages, CCI groups, the presence of preoperative HTN and DM, and preoperative CKD stages.

Considering the surgical and histopathological features of the two groups, high-CONUT-score patients had more advanced pT and pN stages, showed an increased prevalence of venous thrombus, and exhibited a higher Fuhrman grade as compared to low-CONUT score ones. No differences in terms of sarcomatoid features’ presence were found.

### 3.3. Risk Factors Associated with Clinically Significant eGFR Decline

**Figure 2:**
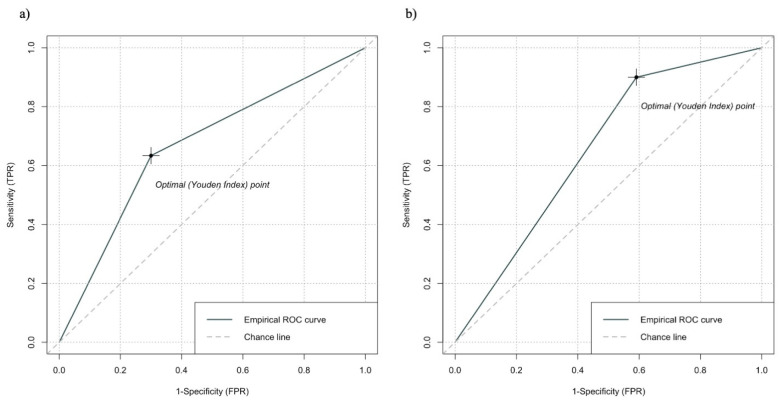
Receiver operating characteristic (ROC) curves for preoperative prediction of clinically significant eGFR decline defined as the development of a stage ≥ IIIb CKD (eGFR < 45 mL/min) at 24 months after RN: (**a**) age ≥ 70 years old, AUC 0.61 (95%CI, 0.51–0.70); (**b**) preoperative CKD stage (II–IIIa), AUC 0.53 (95%CI, 0.43–0.62). Abbreviations are as follows: eGFR: estimated glomerular filtration rate; CKD: chronic kidney disease; RN: radical nephrectomy; AUC: area under the curve; CIs: Confidence Intervals.

At 24 months after surgery, 17 (37.0%) patients in the high-CONUT score and 13 (13.8%) patients in the low-CONUT score groups developed an eGFR < 45 mL/min. A high CONUT score was significantly correlated with the 24-month postoperative eGFR < 45 mL/min (*p* < 0.002). A univariable binomial logistic regression analysis assessing the development of stage ≥ IIIb CKD (eGFR < 45 mL/min) at 24 months after RN is depicted in Table 3. At the univariable analysis, an age ≥ 70 years, preoperative stage II or IIIa CKD, and a high CONUT score were significantly associated with an increased risk of meeting the endpoint (OR 4.03; 95%CI 1.75–9.67; *p* < 0.001), (OR 6.33; 95%CI 2.07–27.6; *p* < 0.001), and (OR 3.56; 95%CI 1.59–8.59; *p* = 0.002). At the multivariable analysis, pre-existing stage II or IIIa CKD (OR 6.05; 95%CI 1.79–28.3, *p* = 0.001) and a high CONUT score (OR 3.98; 95%CI 1.58–10.4; *p* = 0.004) were independently associated with an increased risk of developing stage ≥ IIIb CKD (eGFR < 45 mL/min) at 24 months after RN. No significant contribution of preoperative HTN or DM was found. The AUC values for other predictors included in the MVA model are presented in Figure 2.

## 4. Discussion

The increased risk of postoperative CKD with an eGFR < 60 mL/min is one of the major drawbacks of RN as compared to PN [20]. Although PN, when technically feasible, has become the standard of care in the treatment of localized RCC, RN is still required for several reasons, such as tumor size, location and multifocality, the patient’s comorbidity, and the surgeon’s experience.

In this pilot study, we found that a preoperative high (>2) CONUT score is independently associated with a significant decline in RCC patients undergoing RN.

Some retrospective studies have evaluated the prognostic significance of nutritional status in RCC patients. Elghiaty et al. investigated the prognostic impact of the preoperative CONUT score on survival outcomes (RFS, CSS, OS) in 1046 non-metastatic cT1a-b RCC patients, who underwent RN or PN [13]. A CONUT score >2 was significantly associated with a worse 5-year RFS, CSS, and OS. Similarly, Kang et al. analyzed 1881 patients who underwent RN or PN [14]. In the cT1-3N0 subgroup of 1282 patients, a preoperative CONUT score ≥ 2 was significantly associated with a shorter CSS.

Zheng et al. investigated the prognostic value of CONUT score in 635 non-metastatic RCC patients, who underwent RN or PN, and compared its accuracy with the Prognostic Nutritional Index (PNI), Neutrophil-to-Lymphocyte ratio (NLR), and Platelet-to-Lymphocyte ratio (PLR) as predictors of survival [16]. A multivariate analysis showed that a CONUT score ≥ 2 was an independent predictor of both OS and CSS. Moreover, according to the Hazard Ratios (HRs), the CONUT score outperformed the PNI, NLR, and PLR. Two recent meta-analyses confirmed that a preoperative high CONUT score is able to predict a worse OS and CS in RCC and upper urinary tract urothelial cancer (UTUC) [21,22].

Preoperative counseling before intention-to-cure treatment represents a crucial step in RCC patient management [2,4]. Here, we found that a high preoperative CONUT score >2 is an independent predictor of a clinically significant decline in eGFR after RN for localized RCC. To our knowledge, this is the first study to directly investigate the correlation between CONUT score and renal function. Patients’ age, RN versus PN, and preoperative eGFR are independent predictors of CKD onset and progression, even though their impact on OS still remains controversial [21]. Thus, an eGFR decline after RN or PN represents a surrogate survival outcome in RCC patients and warrants an accurate renal function monitoring and optimization after surgery. Analyzing a multicentric experience evaluating 1213 patients with baseline stage II CKD undergoing either PN or RN, as nephron-sparing surgery was considered to be elective in this group, Hamilton et al. found that those submitted to RN were at increased risk of eGFR decline below 45 mL/min, which was, in turn, associated with a decreased OS [23]. Evaluating surgically induced CKD, Nguyen et al. analyzed 3239 RN and PN RCC candidates. They found that the development of stage IIIb or greater CKD was independently associated with all-cause mortality and non-cancer mortality [24]. Conversely, within a propensity-matched multicenter study, Seung Chung et al. showed that although PN was associated with an improved eGFR compared with RN, it did not yield a significant benefit in survival rates for elderly patients (defined as ≥65 years old) [25].

According to the results of a large multicentric study, approximately 45% of patients recover from preoperative eGFR within 24 months after RN for RCC. Furthermore, eGFR restoration depends on preoperative eGFR; hence, patients with lower preoperative renal function are more likely to recover [26]. Jay et al. demonstrated that such patients have a milder reduction in postoperative renal function at 1 year, due to a greater degree of compensatory structural and functional adaptation after surgery compared to patients with a higher preoperative eGFR [27]. It has been demonstrated that eGFR can increase even 36 months and up to 60 months after surgery, particularly in younger patients without DM and HTN who have a preoperative eGFR ≥ 30 mL/min [19]. Thus, the decision to proceed with nephron-sparing approach, when feasible, should be individualized based on both oncological risk and the probability of a functional decline to IIIb or greater stage CKD. However, further strategies apart from PN need to be considered to prevent a significant eGFR decline. Hence, there is a clinical need to further refine this specific risk stratification.

The comprehensive concordance between the CONUT score and other nutritional scores make it an attractive tool for preoperative detection and the long-term follow-up of malnutrition [12]. The CONUT score is a reproducible, easy-to-use, and cost-effective tool. Ideally it can be managed entirely by a computer and easily integrated into medical reports as an automated process. There are few predictive models available in the literature for postoperative renal function. The first was developed by Sorbellini et al. in 2006 and consisted of a nomogram able to predict the 7-year probability of renal failure according to baseline serum creatinine, ASA score, percentage loss of kidney volume after surgery, and the patients’ age and gender [28]. A recent systematic review by Pecoraro et al. compared 21 predictive models assessing postoperative renal function after surgery for non-metastatic renal tumors (18 studies were included: 9 for PN only; 8 for RN only; 4 for both PN or RN), demonstrating a significant heterogeneity in both model building strategy and reported performance metrics [5]. However, none of these scores investigated the role of immune-nutritional status in post-nephrectomy CKD patients [29]. The incorporation of all the established predictors of postoperative CKD with immune-nutritional status features may lead to the development of a more comprehensive and accurate prediction model. Our study confirms how, in clinical practice, a tool such as the CONUT score might be a useful aid able to identify which patients deserve more attention in regard to renal function preservation during surgical planning or a more intense regimen of supportive care. Moreover, these findings generate the hypothesis that impaired immune-nutritional status, as described by the CONUT score, might be an additive variable worth being investigated in the development of predictive models estimating postoperative eGFR decline after surgical treatment for non-metastatic ccRCC. Furthermore, since poor nutritional status is a potentially reversible condition, it could be of clinical relevance for further studies considering the growing interest in refining and implementing Enhanced Recovery After Surgery (ERAS) protocols.

Our study is not devoid of some limitations, which must be acknowledged. First, its design was retrospective. Moreover, data about smoking status were not available for all patients. Confounding conditions such as drug interaction, including statin therapy, may have affected the CONUT score assessment, leading to a systematic bias. The CONUT score was analyzed as a categorical variable with a predefined cut-off value tailored to this specific cohort of RN candidates. Despite these drawbacks and pending further external validation, this is the first IPD-based multi-institutional experience evaluating the role of the preoperative CONUT score in predicting an eGFR decline in RN patients.

## 5. Conclusions

A preoperative high CONUT score is an independent predictor of a significant eGFR decline in patients with clinically localized RCC undergoing RN. Being easy to use, cost effective, and ideally a completely automated tool, its preoperative assessment could be part of a personalized risk stratification tailored to the clinical conditions and comorbidities of each patient. In light of this, multidisciplinary targeted interventions might be able to improve outcomes by reversing modifiable conditions in order to stem renal function impairment before and after RN. Further studies are pending to draw definitive conclusions.

## Figures and Tables

**Table 1:** Nutrition assessment according to the CONUT score.

Parameter	Normal	Light	Moderate	Severe
Serum albumin (mg/dL)	3.5–4.5	3.0–3.49	2.5–2.99	>2.5
score	**0**	**2**	**4**	**6**
Total lymphocytes (/mL)	>1600	1200–1599	800–1199	<800
score	**0**	**1**	**2**	**3**
Cholesterol (mg/dL)	>180	140–180	100–139	<100
Score	**0**	**1**	**2**	**3**
Screening total score	**0–1**	**2–4**	**5–8**	**9–12**

Abbreviation is as follows: CONUT: Controlling Nutritional Status.

**Table 2:** Descriptive baseline characteristics and the clinicopathological and surgical data of the 140 patients with clinically localized RCC treated with RN according to the CONUT score group.

Variables	Overall	Low CONUT Score	High CONUT Score	*p*
**Patients**, *n* (%)	140 (100.0)	94 (67.1)	46 (32.9)	
**Baseline clinical characteristics**				
**Age (years)**, median (IQR)	66 (56–74)	70 (64–78)	63 (56–73)	0.0005
**Gender**, *n* (%)				0.37
Male	98 (70.0)	63 (67.0)	35 (76.1)	
Female	42 (30.0)	31 (33.0)	11 (23.9)	
**BMI (kg/m^2^)**, median (IQR)	27.0 (25.0–31.5)	26.0 (24.0–31.0)	29.0 (25.0–32.0)	0.12
**CCI**, *n* (%)				0.21
0	71 (50.7)	53 (56.4)	18 (39.1)	
1	38 (27.1)	24 (25.5)	14 (30.4)	
≥2	31 (22.1)	17 (18.1)	14 (30.4)	
**HTN**, *n* (%)	63 (45.0)	38 (40.4)	25 (54.3)	0.17
**DM**, *n* (%)	21 (15.0)	11 (10.6)	10 (23.9)	0.19
**cT stage**, *n* (%)				0.14
cT1 (a, b)	85 (61.2)	64 (68.1)	21 (45.7)	
cT2 (a, b)	34 (21.4)	20 (21.3)	14 (30.4)	
cT3 (a, b, c)	21 (15.0)	10 (10.6)	11 (23.9)	
**Preop. Hb (g/dL)**, median (IQR)	13.7 (12.6–14.6)	13.8 (13.0–14.7)	13.4 (11.6–14.4)	0.55
**Preop. eGFR (mL/min)**, median (IQR)	82.1 (73.1–93.3)	84.0 (71.9–92.1)	82.7 (74.2–93.5)	0.76
**Preop. CRP (mg/dL)**, median (IQR)	0.5 (0.3–2.2)	0.3 (0.3–0.8)	0.74 (0.3–5.83)	0.04
**Fibrinogen (mg/dL)**, median (IQR)	341.0 (282.6–429.0)	325.4 (279.1–390.6)	401.3 (298.2–524.5)	0.007
**Preoperative CKD stage**, *n* (%)				0.31
I	48 (34.4)	31 (33.0)	17 (37.0)	
II	91 (65.0)	63 (66.0)	28 (60.9)	
IIIa	1 (0.7)	0 (0.0)	1 (2.2)	
**Perioperative information**				
**Surgical approach**, *n* (%)				0.52
Open	97 (69.3)	63 (67.0)	34 (73.9)	
Laparoscopic	43 (30.7)	31 (33.0)	12 (26.1)	
**Blood loss (mL), median (IQR)**	100 (50–400)	100 (50–400)	200 (50–500)	0.32
**Intraoperative complications, *n* (%)**	13 (9.3)	5 (5.3)	8 (17.4)	0.04
**Regional LND, *n* (%)**	39 (27.9)	24 (25.5)	15 (32.6)	0.5
**Perioperative complications**, *n* (%)				0.003
None	105 (75.0)	78 (83.0)	27 (58.7)	
Minor	31 (22.1)	13 (13.8)	18 (39.1)	
Major	4 (2.9)	3 (3.2)	1 (2.2)	
**Pathological features**				
**pT stage**, *n* (%)				0.006
pT1 (a, b)	72 (51.4)	55 (58.5)	17 (37.0)	
pT2 (a, b)	23 (16.4)	17 (18.1)	6 (13.0)	
pT3 (a, b, c)	45 (32.1)	22 (23.4)	23 (50.0)	
**pN stage**, *n* (%)				0.5
pN0	39 (27.9)	24 (25.5)	15 (32.6)	
pNx	101 (72.1)	70 (74.5)	31 (67.4)	
**Fuhrman grade**, *n* (%)				0.02
G1	11 (7.9)	11 (11.7)	0 (0.0)	
G2	67 (47.9)	49 (52.1)	18 (39.1)	
G3	48 (34.3)	29 (30.9)	19 (41.3)	
G4	14 (10.0)	5 (5.3)	9 (19.6)	
**Sarcomatoid features**, *n* (%)	4 (2.9)	3 (3.2)	1 (2.2)	0.20
**Necrosis in the specimen**, *n* (%)	65 (46.4)	39 (41.5)	26 (56.5)	0.14
**Venous thrombosis**, *n* (%)	19 (13.6)	8 (8.5)	11 (23.9)	0.03
**Follow-up information**				
**Follow up (months)**, median (IQR)	59.5 (40.0–95.2)	61.0 (39.0–99.0)	54.0 (43.0–92.0)	0.29
**Recurrence events**, *n* (%)	22 (15.7)	11 (11.7)	11 (23.9)	0.11
**All-cause events**, *n* (%)	27 (19.3)	11 (11.7)	16 (34.8)	0.001
**Cancer-specific events**, *n* (%)	12 (8.6)	5 (5.3)	7 (15.2)	0.10
**24-month eGFR < 45 (mL/min)**, *n* (%)	30 (21.4)	13 (13.8)	17 (37.0)	0.002

Abbreviations are as follows: CONUT: Controlling Nutritional Status; IQR: interquartile range; eGFR: estimated glomerular filtration rate; Hb: hemoglobin; CPR: C-reactive protein; RCC: Renal Cell Carcinoma; OR: Odds Ratio; CI: Confidence Interval; DM: Diabetes Mellitus; HTN: hypertension; CCI: BMI: Body Mass Index; Charlson Comorbidity Index; CKD: chronic kidney disease, c/pT/N: clinical/pathological tumor/nodal stage; LND: lymph node dissection.

**Table 3:** Multivariable binomial logistic regression analyses for the prediction of eGFR decline < 45 mL/min among the 140 patients with non-metastatic RCC treated with radical nephrectomy.

	Univariable		Multivariable	
Variable	OR (95%CI)	*p*	OR (95%CI)	*p*
Age (years)				
<70	Ref. (1.0)	-	Ref. (1.0)	-
≥70	4.03 (1.75–9.67)	<0.001	2.01 (1.17–3.09)	0.05
**Gender**				
Male	Ref. (1.0)	-	-	-
Female	2.14 (0.91–4.94)	0.07	-	-
**BMI (kg/m^2^)** as cont.	1.03 (0.91–1.18)	0.6	-	-
**DM**				
No	Ref. (1.0)	-	-	-
Yes	1.58 (0.52–4.36)	0.4	-	-
**HTN**				
No	Ref. (1.0)	-	-	-
Yes	1.29 (0.57–2.92)	0.5	-	-
**CCI**				
0	Ref. (1.0)	-	-	-
1	3.56 (1.41–9.34)	0.07	-	-
≥2	1.46 (0.46–4.39)	0.5	-	-
**Preoperative CKD stage**				
I	Ref. (1.0)	-	Ref. (1.0)	-
II–IIIa	6.33 (2.07–27.6)	<0.001	6.05 (1.79–28.3)	0.001
**cT stage**				
≤70 mm (cT1)	Ref. (1.0)	-	-	-
>70 mm (>cT1)	1.66 (0.73–3.77)	0.2	-	-
**CONUT**				
Low	Ref. (1.0)	-	Ref. (1.0)	-
High	3.56 (1.59–8.59)	0.002	3.98 (1.58–10.4)	0.004
**AUC of the model (95%CI)**			0.80 (0.71–0.88)	

Abbreviations are as follows: CONUT: Controlling Nutritional Status; eGFR: estimated glomerular filtration rate; RCC: Renal Cell Carcinoma; OR: Odds Ratio; CI: Confidence Interval; BMI: Body Mass Index; DM: Diabetes Mellitus; HTN: hypertension; CCI: Charlson Comorbidity Index; CKD: chronic kidney disease, cT: clinical tumor stage; AUC: area under the curve.

## Data Availability

The original contributions presented in the study are included in the article, further inquiries can be directed to the corresponding authors.
